# Citizen Views on an Opt-Out Approach to National Electronic Health Records in Germany: A Small-Scale Qualitative Study

**DOI:** 10.3389/ijph.2024.1607288

**Published:** 2024-07-03

**Authors:** Kimon Papadopoulos, Verena Struckmann, Viktor von Wyl, Felix Gille

**Affiliations:** ^1^ Digital Society Initiative (DSI) and Institute for Implementation Science in Healthcare, University of Zurich, Zürich, Switzerland; ^2^ Technical University of Berlin, Berlin, Germany

**Keywords:** electronic health record, digital health, opt-out, qualitative research, citizen views

## Abstract

**Objectives:**

Electronic health records (German: elektronische Patientenakte - ePA) are an important healthcare tool. However, in Germany, current participation remains low for their national ePA. To rectify this, the German government recently adopted an opt-out approach to their national ePA system. The objective of this study is to investigate and provide a brief overview of German public attitudes towards this approach to inform policymakers with evidence-based insights.

**Methods:**

Four public focus groups were conducted with 12 German citizens to discuss their opinions on the German governments new opt-out approach to the ePA.

**Results:**

Three major thematic categories were identified (Contributors to Opt-Out Implementation, Barriers to Opt-Out Implementation, and Contingent Factors) to describe citizen views on the opt-out approach for the ePA.

**Conclusion:**

The public is generally supportive of an opt-out approach to ePAs in Germany, as they see the benefits ePAs can provide to German society; but they are skeptical on how successful this approach might be due to extant issues that policymakers must be aware of in order to successfully implement an opt-out approach for Germany’s national ePA system.

## Introduction

Electronic health records (German: elektronische Patientenakte - ePA) are instrumental for person-centered healthcare in the digital age in Germany. ePAs contain an individual’s healthcare history in a digital format while allowing their health data to be stored and shared with efficiency and security [1]. With the introduction of ePAs, Germany intends to increase transparency, person-centered care delivery, quality of care, and efficiency, among other benefits [2–5]. In January 2021, Germany introduced an opt-in approach to ePAs, which are run via statutory sickness funds. However, Germany is struggling with the uptake of nationwide ePAs, and participation in ePAs within Germany remains low [3, 6, 7]. As of 2023, less than 1% of German health insurance policy holders have signed up for an ePA [3, 8].

Concurrently, Switzerland is moving from an opt-in approach to an opt-out approach, as less than 0.2% of the population had an ePA in June 2023 [9]. In France, where ePAs are provided with an opt-in approach, only 10% of eligible patients currently have an ePA. However, Austria, which follows an opt-out approach, has nearly 96.9% of its population enrolled in their national electronic health record system [10]. Subsequently, to increase adoption, the German government is changing its implementation strategy from an opt-in solution to an opt-out solution with the target that 80% of statutory insured have an active ePA by the end of 2025 [11]. Contrary to an opt-in model where citizens actively sign-up for an ePA, the proposed opt-out model foresees that citizens receive an ePA by default, unless they actively reject their participation [12]. The opt-out implementation provides a logistically practical legal solution to increasing public participation. Two surveys conducted in 2022 and 2023, respectively, provide indications that the general public support an opt-out approach for the introduction of the ePA [13, 14]. But the switch to opt-out is contested. While adopting an opt-out approach to the use of ePAs will increase public participation in an ePA system, there has been criticism that such an approach will be unpopular amongst the public [8, 15, 16].

The objective of our small-scale qualitative study is to investigate public acceptance of an opt-out enrollment for ePAs in Germany. We aim to provide a summary of public attitudes and expectations to the adoption of an opt-out approach for ePA usage within Germany’s healthcare system and to inform health policymakers with evidence-based insights [17, 18].

## Methods

This study nests in an international comparative study investigating public trust in ePAs, for which we conducted small group interviews (focus groups) to gather general information on German citizens and/or residents’ perception regarding the introduction of an ePA in Germany. Given the topical nature of the opt-out approach in Germany, we decided adhoc to add an additional question about participants’ views and perceptions towards the topic: Aus welchen Gründen würden Sie eine Widerspruchslösung bei der Einführung der ePA in Deutschland unterstützen oder ablehnen? (English: Under which conditions would you support or reject an opt-out introduction of an ePA in Germany?).

The German focus groups were conducted with up to 4 citizens per group, allowing each participant to actively partake and to provide space to express their opinion in depth whilst still reaching saturation by the fourth and final focus group (the most significant indicator of a successful focus group according to previous research) [19–21]. In these focus groups we explored what participants think and how they express their expectations and doubts regarding the use of ePA to one another. For participation in the focus groups, we recruited German speakers living in Germany who were 18 years of age or older. In our sample, saturation was reached by the third focus group, negating the need for further data collection on the matter beyond the four focus groups.

All focus groups were conducted online using Zoom, due to less anticipated risk of drop-out from ongoing public concerns of COVID-19. Additionally, the decision to conduct online interviews was based on our previous qualitative research experience, where there was significantly lower participant enrollment for in-person versus online focus groups. As well, online focus groups allowed for a greater geographic sampling of the German population. We started with recruiting participants for four online focus groups using online flyers and word of mouth. Two to four persons were recruited for each group. The four online focus groups were conducted in February and March 2023. Focus groups were moderated and co-moderated by KP and FG. Focus group discussions were conducted over the course of one and a half hours, with 30 min dedicated to our question on citizen views on Germany’s opt-out approach to their national ePA system. Demographic data of participants is presented in Table 1. Interviews were conducted and transcribed verbatim in German and translated into English.

**TABLE 1:** Participant demographics (Germany, 2023).

Baseline characteristic	Participants (n = 12)	
	*n*	%
**Gender**		
Female	7	58.3
Male	5	41.7
**Age**		
18–29	5	41.7
30–49	3	25.0
50–64	0	0
65+	1	8.3
No Response	3	25.0
**Country of Origin**		
Germany	10	83.4
Lithuania	1	8.3
Not answered	1	8.3
**Current place of residence (Federal State)**		
Berlin	5	41.7
Baden-Württemberg	1	8.3
Bavaria	1	8.3
Brandenburg	1	8.3
North Rhine-Westphalia	4	33.3
**Highest educational level**		
Obligatory school	1	8.3
Vocational training	1	8.3
University degree	10	83.4
**Employment**		
Unemployed	0	0
Student	3	6
Employed	8	16

Participants were on average 32.8 years old (SD, 12.7). Demographic questions were optional, so not all participants provided responses for each question.

Utilizing MAXQDA, a software program designed to assist in the analysis of qualitative and mixed-methods data, FG and KP conducted an independent inductive analysis. FG, a native German speaker, conducted the analysis using the original German transcripts and KP, a native English speaker, conducted the analysis using the translated English transcripts. Discrepancies in translation from German to English were corrected by FG and KP. Following Elo and Kyngäs, 2008, qualitative content analysis process, in an iterative, inductive analysis, both researchers determined, independently, common themes, ideas, and opinions emerging from the data [22]. The two analyses were compared and thereafter combined to create a comprehensive list of common themes present throughout the focus group discussion. Themes were examined and summarized into three different categories. Categories were based upon relevance to an overarching principle, along with conceptual correlation development and work previously done by FG and KP. VS independently reviewed the results in the context of the German healthcare system. Due to the small sample size, no ratings of themes in terms of their relative importance to each other are reported.

A list of health system actors was identified that the public perceive as instrumental in enabling either the success or failure of an opt-out approach to ePA implementation within Germany’s healthcare system.

## Results

Four focus groups, with a total of 12 participants, were conducted. Three major thematic categories were identified: (I) Contributors to opt-out implementation, (II) Barriers to opt-out implementation, and (III) Contingent factors (factors which can either hinder or support an opt-out approach, depending on the context) for the implementation of an opt-out system for the ePA. The list of categories, themes, and sub-themes is shown in Table 2 and are described below.

**TABLE 2:** Influential factors on citizen views towards an opt-out approach (Germany, 2023).

Contributors to *opt-out implementation*	
Themes	Sub-themes
Motivation to participate	Motivation to be involved
	Overcoming laziness (Inertia)
	Time-saving
Societal benefits for all	Benefits to society
	Learning from previous errors (Organ donation approach)
**Barriers *to opt-out implementation***	
**Themes**	**Sub-themes**
Feeling coerced	Coercion to participate (“Silent consent”)
	Lack of autonomy
Fear of loss of control over data	Fear of 3rd party access to collected data
Unmet communication needs	Ignorance of the general public
	Lack of awareness campaigns for ePA
	Education for the public
Socio-political inhibitors	Over-politicization of opt-out approach
	Regulation for secure and confidential data
**Contingent factors**	
**Themes**	**Sub-themes**
Doctor-patient relationship	Physician viewpoint
Method of implementation	User experience of ePA apps
Self-determination	Active vs. coerced participation
Trust in authorities	Health system/government actors

### Contributors to Opt-Out Implementation

1.) Motivation to participate

Participants suggested that an opt-out approach can motivate people to be more involved in using their ePAs. Participants believe that since everyone will be automatically enrolled in having an ePA, they would be more likely to use their ePA. This in turn would create awareness on the individual and societal benefits that the ePA can bring, which would further motivate them to continue participating in the national ePA system. Participants also stated it will engage people who are too “lazy” to opt-in on their own accord, and, by having these people enrolled, it will help combat ignorance to the true nature of ePAs and help build trust between ePAs and the public. As well, it was mentioned that time factors into motivation, as those who are willing to participate in a national ePA system may not want to devote the time to sign themselves up. An opt-out system allows them to conveniently be enrolled without having to invest any of their own time.

*“I think that the process of the state opening* ePAs *and having to appeal is very good at first, because the first consequence of this process would be that everyone would have to deal with this whole issue. And I say, create awareness.”* (FG1)

2.) Societal benefits for all

Another contributing argument is the benefit that ePAs bring to society as a whole. By automatically enrolling the population into a national ePA system, participants expressed that there will be benefits in patient care and future healthcare research for the average citizen. This benefit comes from an increased ease in access to important health information, as well as doctors being more informed on a patient’s healthcare. Many participants also compared the quandary of an opt-out approach to a national ePA system to the organ donation system in Germany. It was stated that if an opt-out approach was instituted for organ donation, it would greatly benefit German society. Many of the participants were convinced that it would be better for society if ePAs were made opt-out instead of opt-in, as they argue that many people are not fully aware of the benefits that ePAs can bring to German society.

*“It is actually a working tool which, from my point of view, benefits everyone.”* (FG1)

### Barriers to Opt-Out Implementation

1.) Feeling coerced

One barrier to implementation, was that of consent. Participants expressed a concern of “silent consent,” where they felt uncomfortable that, in an opt-out system, their consent is given without them explicitly volunteering it. They felt this led to a lack of autonomy and a feeling of coercion, which made them resistant to the notion of an opt-out approach.

*“Personally, I think I would rather reject it because of my experience … I simply forgot about it and then I had to give silent consent … which was not always so good for me.”* (FG3)

*“When I'm forced to do something, I'm more critical of it.”* (FG2)

2.) Fear of loss of control over data

Another barrier brought forth during the interviews was a concern about one’s data having to be entered online. Some participants expressed fear with having their data being uploaded digitally and not being able to remove that data later on if they wanted to do so. This was also part of a larger concern in regards to digital technology and digital health fears.

*“Now if I imagine I'm in a scenario … I do not necessarily want to share … I'm actively uploading something or I get into the stress situation and think, oh my God, how do I get rid of this data from the internet again?”* (FG4)

3.) Unmet communication needs

Issues regarding information flow were also highlighted by focus group participants as a barrier to successful implementation of an opt-out system. Participants stated that they had concerns surrounding how information regarding the opt-out approach and a person’s health data would be communicated to the public and to vulnerable groups; as creating awareness through education to combat perceived ignorance of the general public was seen an important responsibility to be tackled if an opt-out is to be successful.

*“There is very little to nothing … there is no information. I received a letter from my health insurance company about the electronic patient file for the first time … 2–3 weeks ago. …, although this topic has been discussed for many, many years, so it's just too little information and if I do not know anything, what am I supposed to do?” (*FG4)

4.) Socio-political inhibitors

Socio-political issues were amongst the main concerns for participants across all groups that created hesitation in support of an opt-out system. Participants discussed their concerns regarding the increasingly politically volatile topic that they believed was not being discussed in good faith. The main concern was that there was not sufficient regulation in place to protect the security, confidentiality, and autonomy of personal data, where certain health system actors, in particular health insurance companies, may take advantage of people being “coerced” into giving their health data. As well, there were fears that certain vulnerable groups, such as the “technologically illiterate,” in particular those over 65 years old, may be taken advantage of or their rights are curtailed in such a scenario.

*“How is it regulated if someone refuses, especially for vulnerable groups, how is that ensured? That this is not forgotten or somehow overlooked in the process.”* (FG1)

### Contingent Factors

1.) Doctor-patient relationship

The doctor-patient relationship and their communication was rated as a contingent factor by the participants. For the participants, the opinion of their physician holds considerable sway over their own if their relationship is built on trust. They have stated they are more likely to be in support of an opt-out approach if their doctor is.

*“I also think that when I think about it now, that the doctor is also a person I trust … a person approaches me to whom I have a relationship and then I perhaps deal with it more than when my insurance company brings it to me”.* (FG3)

2.) Method of implementation

A second contingent factor mentioned was the method of implementation for ePAs with an opt-out approach. The process in which an opt-out system is implemented can heavily influence the publics perceptions on whether or not they will feel inclined to support such a measure. Participants stated efficient implementation (i.e., convenience), with effective communication and digital health tools from public health stakeholders, will increase public support for an opt-out approach to ePAs. However, the opposite is true if implementation is messy, usage of healthcare applications and portals is hard and/or convoluted, and the rules and regulations of a person’s health data are not conducive to the security and privacy of a person’s data.

*“My personal experience was installing this app on my mobile phone alone. First you have to identify yourself with the electronic identity card and then you can install the main app, but then you have to install an extra app. For the ePA, which then has to be verified again with the main app. …So that's very inconvenient.”* (FG3)

3.) Self-determination

One contingent factor highlighted by participants is the concept of autonomy. Certain participants believe that by implementing an opt-out system for ePAs, their autonomy will be stripped from them due to feeling coerced to participate without being part of the decision-making process. Conversely, others believe that when one decides to opt-out, they have become an active participant by making that choice, and it allows one to exercise their autonomy more actively when compared to an opt-in system.

*“I can imagine that this opt-out solution also takes decisions away from many people.”* (FG3)

*“If you want to contradict it, then you have to actively respond to it. So you have to become active, so to speak, in order to get out of the number.”* (FG 3)

4.) Trust in authorities

Trust also plays a critical role in the publics support for an opt-out approach to an ePA system nationwide. Support, though, is contingent on how much trust the public has in the authorities, institutions, and actors that are in charge of implementing the opt-out system for national ePAs. If trust exists, the public is more likely to be in support of the measure. This also extends to the health system and governmental actors who are responsible for the implementation and adoption of ePAs in the national healthcare system. All actors mentioned by participants that influence trust in the opt-out approach to ePAs are provided in Table 3.

*“I also think that when I think about it now, that the doctor is also a person I trust … a person approaches me to whom I have a relationship … then I perhaps deal with it more than when my insurance company brings it to me in a somewhat generally valid way.”* (FG 3)

**TABLE 3:** Influential system actors, as described by German citizens (Germany, 2023).

Health system actors
Doctors
ePA user interface and user experience designers
Healthcare researchers
Insurance companies
Patients
Policymakers
PR personnel
The public
Vulnerable groups

## Discussion

### Participant Viewpoints

In regard to a national ePA system within the German healthcare system, participants supported an opt-out solution, with caveats, which is reinforced by literature on opt-out approaches to organ donation and utilizing personal health data within ePAs in healthcare research [23–27]. Nevertheless, participants pointed out that barriers to the implementation of an opt-out approach to ePAs exist, and need to be addressed.

Across all focus groups, concern focused on the issues of data privacy and current public knowledge of ePAs. Participants extensively discussed issues of politicization, the technological ineptitude of some population groups, concerns over health data access, a general lack of awareness on the actual function and purpose of ePAs, and that there will be pushback from some members of the population towards being “forced” into having an ePA. These findings line up with another study on patient and physician attitudes towards an opt-out approach to ePAs in Austria [28].

Concerns about a lack of public awareness was the reason why many focus group participants were supportive of an opt-out approach. Participants commented on the failure of the organ donation scheme in Germany to convince the public to participate, which all participants who mentioned it commenting on how that created a detriment to society [23]. Study participants anticipated that by conducting an opt-out approach, even though it will be tough and make some dissatisfied, the missteps of the organ donation scheme can be avoided, and this approach could create greater long term societal benefits than otherwise. Moreover, societal values may change over time and a supportive environment is created during the diffusion process of the ePA.

Through analysis of citizen viewpoints, policymakers can make informed decisions on what approaches citizens trust the most [29]. Additionally, policymakers and stakeholders can find where current approaches lack, while closing gaps and rectifying earlier missteps. By understanding citizen viewpoints, healthcare stakeholders can put forth and create lasting, beneficial change to the population they serve, including ePA implementation [16, 29]. Policy is only truly successful when a population adheres to it and participates in the health intervention(s) put forth. Citizens will only participate and legitimize policy when they feel they are heard and understood, and that they trust that policymakers and healthcare stakeholders have their best interest at heart [16–18, 29].

### Benefits and Limitations of an Opt-Out Approach

Benefits of an opt-out approach are derived from its effectiveness in implementation. One reason is the phenomenon of loss aversion, where the potential of a loss weighs more on decision making than on the potential to gain; as when one deviates from the current standard, the feeling of losing out on what others are gaining can significantly impact public behavior. This phenomenon promotes adherence to common practice, and is also reinforced through the lens of a *status quo* bias [8, 30, 31]. In addition to loss aversion, people often postpone decision-making when it requires an active response, as an active response usually requires time or may include aspects of uncertainty. A final reason for opt-out’s effectiveness is that people also often tend to choose whatever constitutes as the “default option,” as it is assumed that this option was chosen as default for a specific reason; representing an implicit recommendation for the action that coaxes people into a feeling of security and trust [8, 30].

Opt-out approaches have limitations. The first of which is that the decision in an opt-out system leads to feelings of “implied consent,” as they were not actively providing their consent on their participation, which can potentially cause an overall sense of less satisfaction and a lack of commitment with the choice than if the decision was made actively [8, 12]. In addition, opt-out approaches may not accurately reflect the actual preference of the individual making the decision, as if they do not choose to opt-out, it is only assumed that they chose to stay with the default option [8, 30, 32]. Lastly, an opt-out system can be seen as counterproductive to increasing and solidifying participation if policymakers are using an opt-out approach primarily because they view it as the “easier” option; despite the fact there are other interventions that can lead to more considerable results, such as with public-facing educational programs that enable people to have all the necessary information to make an informed decision [8, 30].

### Health System and Governmental Actors

The relationship and amount of trust people have in the actors associated with implementing an opt-out approach to a national ePA system has a great effect on their personal opinions and their support for an opt-out approach. Different research shows that actors’ networks are influential on trust building [33, 34]. The actors shown in Table 3 were explicitly mentioned by participants as actors who can influence public support for opt-out. These actors play a role in placing accountability in the governance of health data, especially within a national ePA system. It has been argued in prior research that an opt-out approach puts the responsibility of administration of information to healthcare organizations [8]. Yet, doctors and politicians held the most prominent significance for trust building in the participants perceptions. Participants were more trusting of doctors and their opinions, while conversely wary of politician’s political motives due to their perceived “over-politicization” of the issue and due to the failure of the organ donation scheme. The relationship between a doctor and their patient is significant due to the privileged rapport a patient has with their doctor. This relationship was noted significantly by participants, and was therefore identified as its own, separate factor within the results.

According to the participants, these actors must take great care and responsibility when implementing an opt-out approach, otherwise trust within the system will be hard to obtain and sustain. Policymakers should ensure that the public’s motivations and critical predictors for adoption are heard and considered. Moreover, potential risks of an opt-out approach need to be kept to a minimum. It is the responsibility of these stakeholders to make sure of this. These results have been supported by findings in previous research where trust in actors, such as physicians and healthcare system stakeholders (both within and outside government), influences people’s perceptions and trust regarding ePAs and an opt-out system [12, 28, 35].

### Limitations

Our study has some limitations, which should create opportunities for future research. One limitation of our study is the small sample size. There was a noticeable lack of individuals aged between 50–64 years. Though three participants did not provide their age, and therefore could be within the 50- to 64-year-old demographic, we cannot say with certainty if any were able to be interviewed for the study. As well, we lacked diversity in terms of educational background. 83.4% of participants had university degrees, yet only 18% of the German population has a university-level education [36].

Interviews with the public who are in the 50- to 64-year-old demographic block and do not have a university degree, along with minority groups and hard to reach communities, are necessary to increase the validity of the findings. Future research should also apply a longitudinal study design to gain further insights into a more diverse group of users and their adoption intentions.

### Conclusion

Through participation in a national ePA system within Germany, the public will gain several advantages, including improvements to quality of care and healthcare research, and a reduction in medical costs and errors. By providing a brief glimpse into the views of everyday German citizens on this issue, we can see that the study participants are generally supportive of an opt-out approach to ePAs in Germany, as they see the benefits ePAs can provide to German society. However, they are skeptical on how successful this approach might be due to issues such as insufficient public information campaigns and communication, socio-political factors, cultural factors, and issues regarding acquisition of consent.

Creating initiatives that focus significantly on conveying the personal and societal benefits of the ePA and improving digital health literacy amongst the population can mitigate the concerns and doubts of German citizens. These interventions can be conducted through the actions of trusted actors, such as doctors, who can help provide these benefits to the public, leading to a successful implementation of an opt-out system for ePAs within Germany.
